# Epidemiology of Tourette Syndrome

**DOI:** 10.3390/brainsci15050426

**Published:** 2025-04-22

**Authors:** Abdullah Yasir Yilmaz, Joseph Jankovic

**Affiliations:** Parkinson’s Disease Center and Movement Disorders Clinic, Department of Neurology, Baylor College of Medicine, Houston, TX 77030, USA; yasir.yilmaz@bcm.edu

**Keywords:** Tourette syndrome, tic disorders, epidemiology, prevalence, incidence

## Abstract

**Background/Objectives:** Tourette syndrome (TS) is a neurodevelopmental disorder, manifested by tics and a variety of behavioral comorbidities that cluster strongly within families, suggesting a combination of genetic and environmental risk factors. The underlying pathophysiology of TS remains to be elucidated. Understanding the incidence and prevalence across different populations provides valuable insights into the etiology and pathogenesis of the condition and aids in the development of effective treatment strategies. **Methods:** A comprehensive literature search was conducted on PubMed covering the period from 1 January 2000 to 1 January 2025. The search used the terms “Tourette syndrome”, “tics”, “tic disorders”, “epidemiology”, “prevalence”, and “incidence”. **Results:** The prevalence of TS is estimated to be about 1% in children and adolescents and approximately 0.01% in adults, with a male-to-female (M:F) ratio of about 4:1. The prevalence of tic disorders is higher in all studies performed in special education populations. **Conclusions:** Despite substantial methodological variability, our review of the literature indicates that TS is a relatively common neurobehavioral disorder, affecting nearly 1% of children, especially boys. Raising global awareness and expanding training in TS should lead to better identification of undiagnosed patients.

## 1. Introduction

Tourette syndrome (TS), named after the French neurologist Georges Albert Edouard Brutus Gilles de la Tourette, is a neurodevelopmental neurobehavioral disorder manifested by multiple motor and phonic tics [1]. Tics usually emerge in early childhood, peaking in severity just before puberty, around ages 8–12, and usually diminish in late adolescence and early adulthood, although tics might persist or re-emerge during adulthood [2,3]. Psychiatric comorbidities include attention deficit hyperactivity disorder (ADHD), obsessive–compulsive disorder (OCD), anger and impulse control problems, sleep disorders, anxiety, depression, and a variety of other behavioral disorders [4,5].

Although the pathophysiology of TS is not well understood, genetic–environmental interaction has been suggested to play an important role in the etiopathogenesis of this child-onset disorder [6,7]. No TS-causative genes have been identified, but based on genome-wide association studies, the heritability of tic disorders has been estimated to be 0.77–0.92, and monozygotic twin studies show a 64.3% concordance [7,8,9,10]. Since environmental factors also may play a role, well-designed epidemiologic studies may provide valuable insights into the etiology of TS. In this study, we comprehensively reviewed the studies published during the past quarter of a century focusing on prevalence, incidence, mortality, and age and sex distribution across different populations.

## 2. Materials and Methods

**Figure 1:**
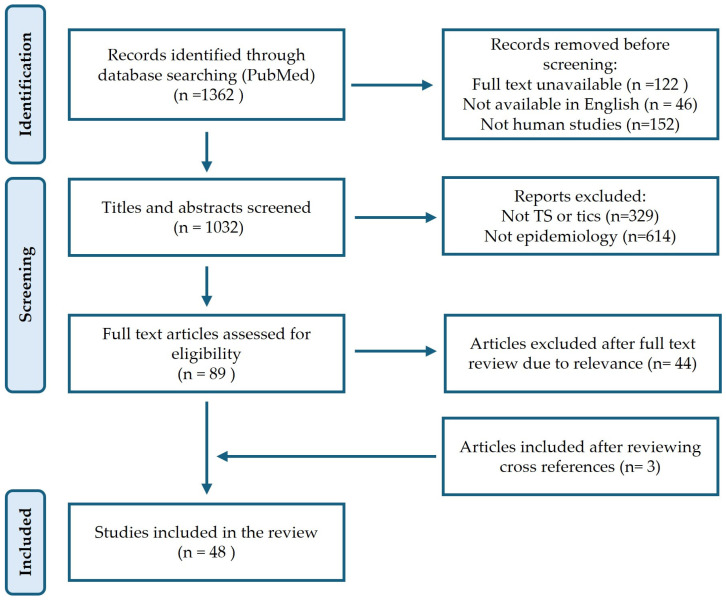
PRISMA flow chart of the article selection for the review.

We conducted a comprehensive literature search on PubMed in January 2025 using the search terms “(Tourette syndrome) AND ((Prevalence) OR (Incidence) OR (Epidemiology) OR (Rate))”. This search yielded 1362 articles (Figure 1). Studies that were unavailable in full text, not in English, not conducted on human subjects, duplicates, or irrelevant to this review were excluded. After screening the titles and abstracts, 89 articles were shortlisted for further evaluation. Following a detailed assessment of the full texts, additional relevant studies were identified and reviewed through cross-references. A total of 48 articles were selected for inclusion in this review.

## 3. Results

### 3.1. Prevalence

A total of 48 studies were reviewed; 27 studies attempted to estimate the prevalence of only TS, 12 focused on the prevalence of all tic disorders (TDs) or chronic tic disorders (CTDs), and 6 studies reported the prevalence of each disorder (Table 1). Studies examining childhood and adolescent populations reported a prevalence of TS ranging from 0.05% to 1.1%. Only two studies assessed the prevalence of TS in childhood and adult populations, reporting estimates ranging from 0.01% to 0.09% [11,12]. The prevalence of tics has been reported to be higher in specific populations. Among individuals with autism spectrum disorder (ASD), an estimated 22% to 33% have a tic disorder, with 4.8% to 12% meeting the criteria for TS and 9% to 12% for CTDs [13,14,15]. On the other hand, 11% of patients with TS were reported to have ASD [16]. Approximately 27% of the special education population exhibits a tic disorder, with TS reported in 1.5% to 7% of this group [15,17,18]. Additionally, one meta-analysis estimated the global prevalence of TS across childhood, adolescent, and adult populations to be 0.5% [19]. This is very similar to the 0.52 prevalence reported earlier, based on a meta-analysis of 21 population-based studies involving children and adolescents [20]. Two other meta-analyses reported the prevalence of TS in children and adolescents ranging between 0.30% and 0.77% [20,21,22]. The estimated prevalence in the adult population was 0.011% [23]. The prevalence trend has shown an increase in recent years in some studies [23], while others have reported no significant changes [24].

The prevalence of chronic tic disorders in the general population has been reported to range from 0.04 to 0.16 [11,25], while in the childhood and adolescent population, estimates range from 0.20% to 0.42% (Table 1). Furthermore, the prevalence of any tic disorder, including transient tic disorders, has been reported to vary widely between 0.03% and 21% in the childhood and adolescent populations [17,26].

**Table 1:** Prevalence of tics and Tourette syndrome.

Study	Country	Ascertainment Database	Disease	Age Group (Years)	Population Size	Number of Cases	%	Prevalence Across Age Groups (%)					Prevalence Across Sexes (%)	
First Author/Year							Total	0 to 2	3 to 5	6 to 11	12 to 17	Adults	Male	Female
Xiong 2024 [24]	USA	NSCH (2016–2022)	TS	0–17	278,472	754	0.23	0.01	0.05	0.28	0.38		0.35	0.11
Mahjani 2023 [27]	Sweden	NBR (1973–2000)	CTD	Up to 40	2,522,677	6227	0.2						0.4	0.1
Bitsko 2022 [6]	USA	NSCH (2016–2019)	TS	3–17	114,476		0.3			0.3	0.4		0.5	0.1
Chou 2022 [25]	Taiwan	NIR (2007–2015)	TS and CTD	All	23,322,805 to 23,758,546	10,103 to 20,001	0.04 to 0.08	Ages 0–18 increased from 0.12 to 0.30 (2007 to 2015); prevalence is 0.51 at age 11				0.02 to 0.04	0.054 to 0.13	0.021 to 0.036
Charania 2022 [28]	USA	NSCH (2016–2017)	TS	6–17	51,001	186	0.3			0.18	0.43		0.44	0.15
Jafari 2022 [19]		Meta-analysis	TS	All	1,136,369		0.5							
Choi 2019 [29]	Korea	NIR (2009 to 2016)	CTD	2–19	20,599		0.26							
Suren 2019 [16]	Norway	NPR (2008–2016)	TS	6–14	556,917	1814	0.43						0.71	0.15
Levine 2019 [30]		Meta-analysis	TS	Adults	2,356,485		0.01							
Yang 2016 [12]	Canada	CCHS (2010–2011)	TS	12+	122,884	122	0.088				0.33	0.066	Adult 0.09	Adult 0.044
Yang 2016 [21]	China	Meta-analysis	TS	3–16	269,571		0.3							
Browne 2016 [31]	Denmark	NBR (1996–2002)	CTD	0–15	89,189	667	0.75							
Yang 2016 [21]	China	Meta-analysis	TD	3–16	269,571		6.1							
Browne 2015 [32]	Denmark	NHR (1980–2007)	CTD	0–18	1,741,271	5596	0.42							
Atladottir 2015 [23]	Denmark	NBR	TS	5–20	1,191,012	3524	0.3	Prevalence between ages 5 and 20 ranged from 0.05 to 0.46%						
	Finland		TS	5–20	1,079,796	1031	0.1	Prevalence between ages 5 and 20 ranged from 0.01% to 0.14%						
	Sweden		TS	5–15	1,797,704	594	0.03	Prevalence between ages 5 and 15 ranged from 0% to 0.07%						
Scharf 2015 [20]		Meta-analysis	TS	4–18			0.54							
Alves 2014 [33]	Brazil	Questionnaire	TS	2–56	9565	42	0.43						0.67	0.22
Bitsko 2014 [34]	USA	NSCH (2011–2012)	TS	6–17	65,540	153	0.19			0.14	0.24		0.32	0.06
Giraldo 2013 [35]	Colombia	A school population	TS	6–12	323	11	3.4							
Knight 2012 [22]		Meta-analysis	CTD				1.61							
Knight 2012 [22]		Meta-analysis	TS	0–18			0.77							
Scharf 2012 [36]	United Kingdom	Population-based birth cohort	TS		7152	13		0.3% (TS narrow) and 0.7% (TS intermediate)						
Kraft 2012 [37]	Denmark	Hospital birth registry	TS	13–15	5974	33	0.6							
Olfson 2011 [38]	USA	Medicaid and private insurance	TS	4–18	10,247,827 + 16,128,828		0.053 to 0.050 in Medicaid and private insurance							
Cubo 2011 [15]	Spain	A school population	TD		1158	125 and 11	16.86% in mainstream and 20.37% in special education population							
Schlander 2011 [11]	Germany	SHI	TD	All	2,238,460	3618	0.16	0–6 years, 0.52		0.79	0.22	0.06 to 0.09	0.19	0.13
Schlander 2011 [11]	Germany	SHI	TS	All	2,238,460	215	0.01	0–6 years, 0.007%		0.04	0.038	0.003–0.009	0.015	0.005
Banerjee 2009 [26]	India	Door-to-door survey	TD	0–19	16,979	6	0.03	0.1% in patients between 10 and 14 years of age					0.06	0.01
Schmid 2008 [39]	Germany	Youth welfare institution	TD	4–18	689	8	1.8							
Simonoff 2008 [13]	United Kingdom	ASD	CTD	10–14	112		9							
Simonoff 2008 [13]	United Kingdom	ASD	TS	10–14	112		4.8							
Stefanoff 2008 [40]	Poland	A school population	TD	12–15	1579	104	6.7	Lifetime prevalence, 9.9%						
Stefanoff 2008 [40]	Poland	A school population	TS	12–15	1579	9	0.6						0.71	0.4
Gadow 2002 [41]	USA	A school population	TD	5–18	3006	246	8.2		22.3	7.8	3.4			
Heiervang 2007 [42]	Norway	A school population	CTD	8–10	996		0.04							
Heiervang 2007 [42]	Norway	A school population	TS	8–10	996		0.16							
Canitano 2007 [14]	Italy	ASD	TS	7–20	105	12	11							
Canitano 2007 [14]	Italy	ASD	CTD	7–20	105	12	11							
Lanzi 2004 [43]	Italy	A school population	TD	6–11	2347	68	2.9						4.4	1.1
Wang 2003 [44]	Taiwan	A school population	TS	6–12	2000	11	0.56							
Khalifa 2003 [45]	Sweden	A school population	TS	7–15	4479	25	0.6						0.9	0.1
Hornsey 2001 [46]	United Kingdom	A school population	TS	13–14			0.76						0.83	0.69
Kurlan 2002 [18]	USA	A school population	TD	8–17	1596	339	21	19.7% in mainstream and 27% in special education population						
Kurlan 2001 [17]	USA	A school population	TS	8–17	1596	15	0.94	0.8% in mainstream and 1.5% in special education population						
Kadesjo 2000 [47]	Sweden	A school population	TS	11	435	5	1.1						1.7	0.5
Hanna 1999 [48]	USA	A school population	TS	6–22	1142	8	0.7							
Costello 1996 [49]	USA	A school population	TS	9–13	1071		0.1						0.13	0.07
Myers 1995 [50]	USA	Individuals with Down syndrome	TS	All	425	5	1.2							
Apter 1993 [51]	Israel	Military screening population	TS	16–17	28,037	12	0.43						0.49	0.31
Wong 1992 [52]	China (Hong Kong)	A school population	TS	7–11	718	3	0.4						0.8	-
Nomoto 1990 [53]	Japan	A school population	TD	4–12	1218	3	5.1							
Nomoto 1990 [53]	Japan	A school population	TS	4–12	1218	3	0.5							
Comings 1990 [54]	USA	A school population	TS	5–14	3034	14	0.46						1.05	0.13
Caine 1988 [55]	USA	A school population	TS	5–18	142,636	41	0.03							
Burd 1986 [56]	USA	Hospital registry	TS	6–18	140,580	73	0.05						0.093	0.01

ASD: autism spectrum disorder; CCHS: Canadian Community Health Survey; CTD: chronic tic disorder; NPR: Norwegian Patient Registry; NBR: National Birth Registry; NIR: National Insurance Registry; NSCH: National Survey of Children’s Health; SHI: Statutory Health Insurance; TS: Tourette syndrome; TD: tic disorder.

### 3.2. Incidence

There is a limited number of studies investigating the incidence of tic disorders, and the available studies exhibit considerable heterogeneity in terms of methodologies, assessed age groups, monitoring periods, and population sizes. The annual incidence of all tic disorders in the general population has been reported to have a statistically significant increase from 0.017 to 0.40 per 100,000 (from year 2003 to 2020) indicating an upward trend [57]. It is not clear whether the observed growing incidence represents a true increase in the frequency of tic disorders or increased awareness and diagnosis.

Interestingly, the reported incidence of tic disorders among children and adolescents appears to vary from one geographic region to another. For example, the incidence of tic disorders has been reported to be relatively low in Asia, ranging from 0.05% to 0.14% in Korea. It also seems relatively low in Scandinavian populations, ranging from 0.02% to 0.1% in the Danish and Finnish populations, respectively [57,58,59].

The cumulative incidence of TS and CTDs in the general population has been estimated at 0.27% by age 41 years in a Swedish study [60]. One study reported the cumulative incidence of TS at 0.3% by 15 years in Denmark [23].

### 3.3. Age and Sex Distribution

Of the 48 studies reviewed, 37 focused on patients under 18 years of age, including 28 studies on TS and 14 on CTDs and/or TDs. Three studies assessed the entire population for TS, while another three evaluated the entire population for CTD and/or TD.

The reported prevalence of TS varied widely across age groups, ranging from 0.007% to 0.05% in children under 6 years, 0.04% to 0.28% in those aged 6 to 11 years (though one study from Brazil reported a notably higher prevalence of 3.4% in a school population aged 6–12 years), and 0.19% to 0.54% among individuals aged 12 to 18 years. The substantial variation in prevalence estimates reflects differences in study methodologies and age groups studied (Table 1).

The male-to-female ratio is reported to be between 2:1 and 9:1, but mostly around 4:1 in childhood and adolescent populations; the male predominance decreases with age [61].

### 3.4. Mortality

All-cause mortality is significantly elevated in the TS population, with an 86% increased hazard ratio for both natural and unnatural causes [62]. TS/CTDs are associated with increased substance and alcohol use and misuse-related outcomes, including alcohol-related disorders and substance-related death [63]. One study reported a mortality rate ratio of 2.30 even after excluding individuals with comorbid ADHD, OCD, and substance use disorders [64].

Patients with TS/CTDs are at an increased risk of self-injurious behavior, involvement in bullying behaviors, and suicide [65]. Approximately 17% of patients exhibit self-injurious behavior [66], and they have an approximately four-fold increased risk of attempting or dying by suicide when compared to healthy controls [67]. The risk of transport-related injuries or death is 1.5- to 1.84-fold higher in patients with tics than in the general population, though this risk loses significance after adjusting for ADHD, suggesting the importance of the comprehensive assessment and management of comorbid conditions [62,68].

## 4. Discussion

In this study, we reviewed all the published prevalence and incidence studies on TS and other tic disorders. The prevalence of TS has been reported to range from 0.03% to 3.4% in children and adolescents and from 0.03% to 0.01% in adults. Meta-analyses estimate the prevalence to be between 0.30% and 0.77% in children and adolescents and approximately 0.01% in adults. It is estimated that TS affects between 350,000 and 450,000 children and adults in the United States [6]. However, a high level of heterogeneity exists between studies, driven by methodological differences and challenges related to diagnostic criteria; sources of information; study design; and demographic factors such as age, sex, race, socioeconomic status, and cultural differences, which may influence healthcare access and the threshold for seeking medical attention.

The prevalence of TDs has been reported to range between 0.035 and 21% in different studies [17,26]. The first study was conducted as a door-to-door survey with field workers, where potential cases identified through questionnaires were subsequently evaluated by a neurologist [26]. Notably, no phonic tics were documented, no prevalence differences between sexes were observed, and the diagnosis relied solely on parental reporting. In contrast, the second study evaluated a school-based population, where trained technicians assessed participants after obtaining parental consent, with each child being observed for 60 to 150 min during the interview [17]. The prevalence reported in the first study was likely lower due to its reliance on parental reporting alone, whereas the second study may have overestimated prevalence, as parents of children with tics were more likely to provide consent for comprehensive evaluation. These methodological differences demonstrate how prevalence estimates of TS can vary significantly depending on the study design. The mean age and male-to-female ratio of the sample are critical factors that can influence the reported results [48].

Some studies have reported that the prevalence and incidence of tic disorders have increased over time [23,57]. This is consistent with a reported increase in the use of mental health services. For example, among children in Finland, the mental health services between 1989 and 2013 increased from 2.4 to 11.0% [69]. This increase may not necessarily be indicative of the growing frequency of tics and TS but a greater awareness of these disorders among patients and physicians [70]. Other studies have shown that one in five adolescents in the US receives mental healthcare, and this proportion remained consistent between 2005 and 2018 [71]. Similarly, the estimated prevalence of TS was stable from 2016 to 2022 [24].

Differentiating tics from myoclonus, dystonia, or functional movement disorders can be challenging, leading to both underdiagnosis and overdiagnosis, further affecting the accuracy of patient diagnosis and prevalence [72,73]. This highlights the importance of and need for specialists trained in TS.

The included studies span a long period, from 1986 to 2024, introducing additional variability in reported prevalence estimates due to evolving diagnostic criteria, including different versions of the DSM, as well as nearly four decades of environmental changes and potential genetic variability within populations over time. There was a recent attempt to refine the diagnostic criteria for tic disorders, but it is not clear why the new definition requires that the tics must “have caused some degree of impairment” as many patients may not find their tics “impairing” or even troublesome [74].

### 4.1. Age

Tics typically have an onset between the ages of 4 and 6 with peaking intensity around 10–12 years. Tic severity decreases during adolescence; about three-quarters of patients improve, and one-third will be tic-free by adulthood, although these proportions are controversial, and most patients may continue having at least mild tics (50–80% of patients over age 16) [3,4,75]. The reported prevalence ranges from 0.04% to 3.4% in children aged 6–12 years and from 0.04% to 0.76% in adolescents aged 12–18. The partial or complete remission that occurs in most patients in their early 20s may be transient, and many patients experience a recurrence or exacerbation of their tics in late adulthood or even when elderly. Although the vast majority of patients with “adult-onset tics” experience a recurrence of their childhood-onset tics, many may not recall having tics during childhood or may not have received a formal diagnosis. In one study of adult patients, with a mean age of 58 years, approximately 80% had a history of tics that began before age 18, with a mean age at onset of 8.5 years [3]. A substantial portion of individuals with TS may remain undiagnosed during childhood and may only receive a diagnosis if they experience an exacerbation that necessitates medical evaluation in adulthood. This diagnostic delay further contributes to the underestimation of TS prevalence, particularly in studies relying on national insurance databases or medical registries.

Approximately 85% of individuals with TS have at least one psychiatric comorbidity, and 57% have two or more psychiatric disorders [76]. ADHD typically has an onset around age 5, followed by OCD at approximately age 7, with the worst OCD symptoms occurring about two years after the peak severity of tic symptoms [77]. Anxiety disorders also tend to emerge around age 7, while mood disorders commonly develop around age 13 [76,78]. The severity of comorbidities, including OCD and ADHD, also significantly decreases with age, although subclinical symptoms often remain [4,78].

### 4.2. Sex

Tourette syndrome is more common in males, despite the highly variable methodologies across studies. The reported male-to-female ratio (M:F) ranges from 1.58:1 to 9.0:1, with meta-analyses estimating a ratio of 4:1 during childhood, which decreases to 2.33:1 in adulthood. Studies have shown that females with TS tend to have a later age of symptom onset and a longer delay between symptom onset and diagnosis [79].

The severity and complexity of tics are generally reported as worse in males during childhood and adolescence, whereas females have a greater likelihood of experiencing tics worsening with aging and worse impairment from tics [61,80,81,82]. Furthermore, the age-related decline in TS prevalence is less pronounced in females than in males, which accounts for the decreasing male-to-female (M:F) ratio with age [21,83]. Females are diagnosed at later ages than males, although the data are mixed; some studies have also reported a later age of onset for tics [76,84], while others report no difference.

Comorbid ADHD and ASD are more common and severe in males with TS compared to females. However, this difference diminishes over time and disappears by adulthood, with no significant differences observed after age 18 [81].

There are controversial results in studies on the prevalence of OCD across sexes in patients with TS [4,76,80]. However, early-onset and late-onset OCD differ in their association with tics and TS. The early-onset OCD group shows higher rates of TS, and the proportion of males is significantly higher compared to the late-onset group [85].

The etiopathogenesis underlying the observed sexual dimorphism in TS epidemiology remains to be fully elucidated. Structural and physiological differences in the central nervous system across sexes may contribute to this disparity. These include sex-specific differences in dopamine receptor expression [86]; variations in striatal regulatory interneurons, which are implicated in the pathophysiology of TS [87,88]; and various anatomical differences, including sensorimotor cortical thinning and putaminal asymmetry [89]. Furthermore, striatal dopamine release is modulated by sex hormones such as estradiol and progesterone; further, vesicular monoamine transporter 2 (VMAT2) protein expression, a therapeutic target in TS treatment, is also regulated by estradiol [90]. Androgens may exacerbate tic severity, suggesting that differential hormonal exposure during early neurodevelopmental periods could contribute to sex-based differences in TS presentation [91]. Longitudinal fluctuations in sex hormone levels throughout childhood, puberty, adolescence, and adulthood may contribute to both clinical manifestations and the epidemiological patterns observed in TS [91].

The higher prevalence of comorbid conditions such as ASD and ADHD in males has been proposed to reflect earlier diagnostic recognition in this group, which may partially account for the elevated M:F ratio that declines with age [80].

### 4.3. Social and Demographic Determinants

TS is present in all populations worldwide, and the literature from Europe and North America reports a higher prevalence compared to other regions [92]. The highest reported prevalence in a general school population was 3.4% in Colombia, followed by 1.1% in Sweden [35,47]. In contrast, the lowest prevalence rates were reported in Germany (0.01%) and the United States (0.05%) [11,56]. As previously discussed, methodological differences across studies—including variations in diagnostic criteria, sampling strategies, and population demographics—substantially affect reported prevalence estimates. A recent meta-analysis reported prevalence in America, Asia, and Europe as 0.6%, 0.3%, and 0.4%, respectively [19].

The reported prevalence of TS was thought to be lower in African Americans and sub-Saharan black Africans compared to other populations [93]. Earlier studies found that the prevalence of TS was nearly twice as high in non-Hispanic White individuals compared to Hispanic and non-Hispanic Black individuals [34]. However, recent studies indicate no significant difference in prevalence between non-Hispanic White individuals and other racial groups [28]. Furthermore, the prevalence of TS among Black individuals has been reported to range from 0.78% to 1.95%, which is comparable to the general population [94]. It has been suggested that a substantial number of children with TS in Africa remain undiagnosed, likely due to limited clinical awareness, insufficient attention to the condition, and low help-seeking behaviors [95].

Studies comparing patients with TS from different regions have found similar tic severity, age at tic onset, sex distribution, and rates of ADHD and OCD but different rates of coprolalia, comorbid oppositional defiant disorder (ODD), conduct disorder, and mood symptoms [96,97]. These findings suggest that while the core phenomenology of TS is similar across cultures, which indicates a shared biological and genetic basis, variations in associated behaviors reflect cultural influences on behavior and clinical presentation [98], as well as perception of impairment and quality of life [99,100].

Some studies have suggested that lower socioeconomic status is associated with a two-fold increased risk of TS and chronic tics [101], while others report no difference in socioeconomic distribution among patients with TS and co-morbid behavioral problems [102] but note higher tic severity in unemployed individuals [103]. One study showed increased comorbidity rates, including ADHD, disruptive behavior, depression, and higher rates of antipsychotic medication use in children under Medicaid compared to privately insured youth [38]. On the other hand, adults with TS have higher odds of earning below the median income and having lower employment status, which may also confound these findings [12].

### 4.4. Co-Morbidities

Comorbid mental, behavioral, and developmental disorders are observed in over 80% of patients with TS, and approximately 60% of the TS population has two or more comorbid psychiatric diagnoses [28,76]. Neurodevelopmental disorders are particularly prevalent, with approximately 50% of individuals with TS diagnosed with comorbid ADHD, 33% with a learning disability, and 10% with ASD [13,16,28]. Schizophrenia has been reported in 1.2% to 7% of patients with TS [29,104]. Although this wide range may be attributed to differences in study populations, diagnostic criteria, and methodologies, the relative risk compared to the general population is consistently reported to be about seven- to ten-fold higher [29,38]. Mood disorders are also common, with anxiety affecting 15% to 60% and depression present in 20% of patients, representing a five- to six-fold increased risk compared to the general population [28,29].

OCD is among the most frequently reported and clinically significant comorbidities in TS. Patients with TS and comorbid OCD exhibit higher levels of disability and greater symptom severity compared to those without OCD [76,104]. The prevalence of OCD in patients with TS ranges from 7% to 50%, representing a seven-fold increase compared to individuals without tics [16,38,76]. Furthermore, approximately 10% of patients with TS present with both ADHD and OCD [36]. However, the severity of both ADHD and OCD symptoms, like tics, also tends to decline over time [4].

Approximately 17% of individuals with TS experience self-injurious behavior, and a meta-analysis has reported rates as high as 35% [66,105]. However, only about 2% of these behaviors are directly related to tics, while 11.4% involve deliberate self-directed harm. The presence of complex motor tics, comorbid OCD, and greater overall tic severity have been associated with an increased likelihood of self-injurious behavior in individuals with TS [66,106]. Although the underlying pathophysiology of self-injurious behavior in TS remains unclear, several mechanisms have been proposed, including deficits in GABAergic inhibitory control; impaired impulse regulation; co-occurring intellectual disability; and the abnormal central processing of noxious stimuli, which may contribute to the development of SIB in this population [66,105,106].

About one-third of patients with TS have comorbid movement disorders other than tics, including chorea-like piano-playing movements (chorea-minima), stereotypies, tremors, dystonia, and parkinsonism [107]. Functional tic-like behaviors (FTBs) are also common comorbidities in patients with TS [108,109]. The prevalence of FTBs in the general population has been reported as 0.16% [108]. FTBs are more frequent in females, usually with comorbid OCD and self-injurious behaviors [109,110,111]. It is, however, important to point out that even experts may have trouble distinguishing TS-related tics from functional tics [112].

Sleep disorders affect up to 65% of individuals with TS [113]. Commonly reported sleep disorders include insomnia, parasomnias such as night terrors and bruxism, and increased motor activity during both REM and non-REM sleep, which may reflect underlying hyperarousal [114,115]. The persistence of tics during sleep and periodic limb movements are frequently observed in this population [116].

Patients with TS and CTDs have a two-fold increased risk for metabolic and cardiovascular conditions, including obesity, type 2 diabetes, and circulatory system diseases [60].

### 4.5. Special Populations

TS is more common in individuals with autism spectrum disorder (ASD). Studies have reported a TS prevalence of 4.7% to 11% and a chronic tic disorder prevalence of 9% to 11% among individuals with ASD [13,14]. Another study showed TS prevalence in the special education population as twice that of the regular education population with prevalences of 1.5% and 0.8%, respectively [18]. On the other hand, the prevalence of ASD among individuals with TS under the age of 17 is reported to be 21%, compared to 2.75% in the general population of the same age group in the USA [24] and 11% in the Norwegian TS population [16].

One study reported a TS prevalence of 1.6% in individuals with Down syndrome at a child development center; although these patients were on neuroleptic treatment and had a relatively late onset of tics, the possibility of tardive syndrome rather than TS cannot be ruled out [50]. Given that behavioral difficulties such as oppositional defiant disorder, learning disabilities, developmental delays, speech and language disorders, intellectual disabilities, and self-injurious behaviors associated with co-morbidities often present more prominently for affected individuals and their families, these challenges can mask tics and delay or complicate the diagnosis of Tourette syndrome [24,70]. Therefore, careful phenomenological evaluation in this population is crucial.

## 5. Limitations

The primary limitation of this study is the significant methodological heterogeneity among the included studies, including variations in sample size, study design, and the age groups assessed. These differences limit comparability and contribute to the wide range of reported prevalence estimates, thereby reducing their accuracy. Furthermore, while populations in the United States; Scandinavia; and parts of Asia, including China and Taiwan, have been well studied, data on TS and TD prevalence remain scarce in other regions. This geographic and demographic imbalance limits the generalizability of the findings. Additionally, as previously discussed, the presence of undiagnosed and undetected cases remains a significant barrier to accurately determining the true prevalence of TS. Another limitation is that the literature search was restricted to articles published in English and indexed in PubMed, which may have reduced the number of studies assessed and introduced potential publication bias.

## 6. Conclusions

The reported prevalence of TS varies considerably, ranging from approximately 0.30% to 0.77% among individuals under 18 years of age and around 0.01% in the general population. The marked heterogeneity across studies, driven by methodological differences and demographic variations, contributes to the wide range of reported prevalence estimates. Age; sex; social determinants such as healthcare access and cultural context; and high rates of comorbidities, including ADHD, ASD, and OCD, greatly influence the clinical course and reported epidemiology of TS. Given the possibility of a substantial number of undiagnosed cases, large-scale, population-based studies are essential for obtaining more accurate prevalence estimates. Increasing public awareness and improving specialist training in the diagnosis and management of TS are important steps toward addressing this gap.

## Abbreviations

The following abbreviations are used in this manuscript:

TS	Tourette syndrome
TD	Tic disorder
ADHD	Attention deficit hyperactivity disorder
OCD	Obsessive–compulsive disorder
ASD	Autism spectrum disorder
M:F	Male-to-female ratio
CTD	Chronic tic disorder
DSM	Diagnostic and Statistical Manual of Mental Disorders
ODD	Oppositional defiant disorder
